# Association of physical activity with future mental health in older, mid-life and younger women

**DOI:** 10.1093/eurpub/ckt199

**Published:** 2014-02-14

**Authors:** Amanda Griffiths, Anne Kouvonen, Jaana Pentti, Tuula Oksanen, Marianna Virtanen, Paula Salo, Ari Väänänen, Mika Kivimäki, Jussi Vahtera

**Affiliations:** 1 Division of Psychiatry and Applied Psychology, School of Medicine, University of Nottingham, Nottingham, UK; 2 School of Sociology, Social Policy & Social Work, Queen’s University Belfast, Belfast, UK; 3 UKCRC Centre of Excellence for Public Health (NI), Queen’s University Belfast, Belfast, UK; 4 Finnish Institute of Occupational Health, Helsinki and Turku, Finland; 5 Department of Psychology, University of Turku, Turku, Finland; 6 Department of Epidemiology and Public Health, University College London, London, UK; 7 Institute of Behavioral Sciences, University of Helsinki, Helsinki, Finland; 8 Department of Public Health, University of Turku and Turku University Hospital, Turku, Finland

## Abstract

**Background:** Mental ill-health, particularly depression and anxiety, is a leading and increasing cause of disability worldwide, especially for women. **Methods**: We examined the prospective association between physical activity and symptoms of mental ill-health in younger, mid-life and older working women. Participants were 26 913 women from the ongoing cohort Finnish Public Sector Study with complete data at two phases, excluding those who screened positive for mental ill-health at baseline. Mental health was assessed using the 12-item General Health Questionnaire. Self-reported physical activity was expressed in metabolic equivalent task (MET) hours per week. Logistic regression models were used to analyse associations between physical activity levels and subsequent mental health. **Results**: There was an inverse dose–response relationship between physical activity and future symptoms of mental ill-health. This association is consistent with a protective effect of physical activity and remained after adjustments for socio-demographic, work-related and lifestyle factors, health and body mass index. Furthermore, those mid-life and older women who reported increased physical activity by more than 2 MET hours per week demonstrated a reduced risk of later mental ill-health in comparison with those who did not increase physical activity. This protective effect of increased physical activity did not hold for younger women. **Conclusions**: This study adds to the evidence for the protective effect of physical activity for later mental health in women. It also suggests that increasing physical activity levels may be beneficial in terms of mental health among mid-life and older women. The alleviation of menopausal symptoms may partly explain age effects but further research is required.

## Introduction

Mental ill-health, particularly depression and anxiety, is a leading and increasing cause of disability. Unipolar depressive disorder alone is currently the fourth leading cause of disease burden worldwide. Predictions suggest it will become the largest by 2020.^1^ The prevalence of mental ill-health is a matter of particular concern for women.^2^

There is growing evidence from developed countries that physical activity is associated with better mental health. The strength of evidence has been limited by cross-sectional designs and small, homogenous or clinical samples. Many studies have failed to take into account baseline mental health, pre-existing major disease and other potentially confounding variables. In studies involving non-clinical samples, careful measurement of symptoms of mental ill-health is important since such problems are often characterized by shifting patterns of symptoms that resist the precise classification used for clinical populations.^3^ However, conceptually varied assessments of mental health have been employed, including psychological distress, psychological wellbeing, quality of life, depression and anxiety. Many studies use estimates of time spent on physical activity rather than estimates of energy expenditure. Finally, many studies have short-term follow-ups, often of <12 months.^4^

Nonetheless, reviewers have concluded that there is reasonable evidence from prospective, methodologically robust studies, for an inverse relationship between level of physical activity and mental health^4–6^ although the detail of a dose response relationship is not yet entirely clear.^7^^,^^8^ One of the more robust studies, involving solely mid-life women, after adjusting for potential confounders such as educational status, occupation, body mass index, smoking, baseline depression and existing major disease, found a clear dose–response relationship between increasing physical activity and decreasing depressive symptoms.^9^

Exploring the effects of change in physical activity levels over time may provide additional insights to those provided by reports of habitual activity, although possibly more reliably if those changes are sustained.^10^ Results from a prospective general population survey showed that women whose physical activity levels decreased from active to inactive had a 51% greater probability of developing depression compared with women who remained active.^10^ A study of mid-life and older nurses found that those with low-physical activity levels at baseline who later increased activity levels to recommended levels, demonstrated subsequent improvements in mental health.^11^ And a study of mid-life women found a decreased risk of later depression for those who were in the lowest category of physical activity level at baseline but who increased to recommended levels.^9^

Recommended levels of physical activity for adults have been published since the mid-1990s. Guidelines from the UK, USA and the World Health Organization (WHO) recommend at least 150 min of moderate intensity aerobic physical activity, or 75 min of vigorous intensity physical activity, throughout the week, for periods of a minimum of 10 min. They also advise muscle-strengthening exercises on at least 2 days per week.^12–14^ The WHO recommends 300 min of moderate intensity physical activity per week for additional health benefits.^14^ This higher level meets current recommendations for healthy weight maintenance^15^ and cancer prevention.^16^ There is insufficient evidence to inform the precise amounts of physical activity needed for such benefits to mental health and well-being.^4^ With regard to depression in women there is promising evidence that low levels of physical activity (<150 min week^−^^1^) can be beneficial.^7^ In the case of mid-life women, it has been suggested that even very low levels (<60 min a week) may be protective.^9^ More precise quantification of recommended physical activity levels to prevent symptoms of mental ill-health would help develop public health recommendations and health promotion campaigns.^9^

### Aim of this study

The purpose of this prospective study was to explore the relationship between physical activity and symptoms of mental ill-health in a large, well defined and heterogeneous sample of working women. It aimed in particular to explore the existence of a dose–response relationship and to investigate the effects of increased physical activity for subsequent mental health in younger, mid-life and older women.

## Methods

Data were derived from the Finnish Public Sector Study (FPSS), an ongoing cohort study involving all personnel employed from 1991/2000 to 2005, from 10 municipalities and 21 hospitals in Finland. In this study, we used repeated survey data of those who responded at Phase 2 collected in 2000–02 (*N* = 48 598; response rate 68%) and Phase 3 collected in 2004–05 (*N* = 35 914; response rate 77% of those who responded at Phase 2) or at Phase 3 and Phase 5 collected in 2008–09 (*N* = 36 721; response rate 78% of those who responded at Phase 3) of FPSS. If the participant had responded at all phases, Phase 3 and Phase 5 responses were used. The total sample size was 48 273 participants, 39 446 of whom were women. The analyses in this study involved 26 913 women (mean age 45.6 years; SD = 9.8; range 18–69 at baseline) after excluding women who by General Health Questionnaire screened positive for mental ill-health at baseline (*N* = 10 145) or had incomplete data on any study variables (*N* = 2388). The final study cohort did not substantially differ from those women who did not respond to the follow-up survey in terms of mean age (45.6 years in the sample vs. 41.8 years in the drop out population) or occupational position (13% manual vs. 17% manual).

Using personal identification numbers (the unique number assigned to all Finnish citizens that documents date of birth and gender), the respondents were linked to comprehensive national health registers from 1994 to 2005 to obtain data on some covariates. The study was approved by the Ethics Committee of the Hospital District of Helsinki and Uusimaa.

### Physical activity

Participants reported the average weekly amount of time spent on physical activity (including the journey to and from work) corresponding to the intensities of walking, vigorous walking, jogging and running. The time spent in each type of activity, expressed in hours per week, was multiplied by its typical energy expenditure, expressed in metabolic equivalent tasks (METs). One MET is the caloric need per kilogram of body weight per hour of activity, divided by the caloric need per kilogram per hour at rest: the energy required while sitting quietly. The total physical activity score for each participant was expressed as the sum score of MET hours per week.^17^ It is thus a measure of how much energy is expended on physical activity, rather than the more commonly used measure of time spent on physical activity.

We categorized physical activity into four groups: <14 MET hours per week (low), 14≥ MET hours per week <30 (intermediate), 30≥ MET hours per week <60 (high) and ≥60 MET hours per week (very high). Our categorization of MET scores maps onto contemporary recommended levels of physical activity. At least 14 MET hours per week (equivalent to 150 min of moderate intensity activity, such as brisk walking) corresponds to the recommended minimum energy expenditure for reducing most known health risks associated with inactivity^18^ and a minimum of 30 MET hours per week meets current recommendations for healthy weight maintenance^15^ and cancer prevention.^16^

We measured change in physical activity levels between Time 1 (2000 and 2004) and Time 2 (2004 and 2008). Where physical activity had increased or decreased by more than two MET hours per day, this was coded as ‘increased physical activity’ or ‘decreased physical activity’, respectively. Where physical activity had changed by less than two MET hours per day in either direction, this was coded as ‘no change’ and used as the reference group.

### Mental health

Mental health was assessed using the 12-item General Health Questionnaire (GHQ-12). This has been widely used in many languages and settings in population-based research.^19^ The GHQ-12 measures both positive and negative aspects of mental health.^20^ Each item inquires as to whether the respondent has experienced a particular symptom or behaviour recently. Responses are scored as either 1 or 0 to indicate the presence or absence of a symptom. We defined women with a total score of 4 or more as scoring positively for mental ill-health.

### Covariates

Baseline covariates obtained from employers’ registers included occupational position, based on the occupational-title classification of Statistics Finland^21^: high (e.g. teachers, physicians), intermediate (e.g. registered nurses, technicians) and low (e.g. cleaners, maintenance workers). Information on marital status (married or cohabiting vs. other) and night/shift work (yes vs. no) was obtained from the survey. Standard questions were used to assess heavy drinking (>210 g week^−1^ vs. less) and smoking status (current smoker vs. non-smoker). Self-reported height and weight were used to calculate body mass index.

The presence of chronic illness was derived from the Finnish Drug Reimbursement Register which contains information on persons entitled to reimbursement for the treatment of chronic conditions and diseases, and the date when the reimbursement is granted. Patients who apply for reimbursement have to submit a detailed medical statement from their treating physician confirming the diagnosis. We identified all participants with hypertension, cardiac failure, ischaemic heart disease, diabetes, asthma or other chronic obstructive lung disease and rheumatoid arthritis at the end of the baseline survey year. Data on cancer diagnosed during the baseline survey year or four preceding years were obtained from the Finnish Cancer Registry which compiles all notifications of cancers nationwide.^22^ The presence of any of these illnesses was coded as yes or no.

### Statistical analysis

Logistic regression models were used to analyse the associations between levels of physical activity at baseline and the likelihood of mental ill-health at follow-up in those participants who did not screen positive for mental ill-health at baseline. The reference category comprised participants reporting low levels of physical activity (<14 MET hours per week). In addition, logistic regression analysis was applied to examine the association between change in physical activity levels between Time 1 and Time 2 and the likelihood of mental ill-health at Time 2. Analyses were performed separately for three age groups: 18–44 years (younger women), 45–54 years (mid-life women) and 55–69 years (older women). Statistical models were first adjusted for age, occupational position and marital status; then additionally for night/shift work, heavy drinking, smoking, body mass index and chronic illness. We analysed all occupational position categories together and adjusted the models for occupational position since the occupational position interaction was not significant (*P* = 0.9). All analyses were performed with SAS version 9.2 statistical software (SAS Institute Inc., Cary, NC, USA).

## Results

The characteristics of the study sample are shown in table 1. The majority of participants (76%) were married or cohabiting and 60% were categorized as being of intermediate occupational position. A total of 4666 (17%) participants scored positively for mental ill-health during follow-up. The incidence of later mental ill-health was higher in participants below age 45 at baseline, in smokers and in heavy drinkers.

**Table 1:** Baseline characteristics of the participants and the incidence of mental ill-health at follow-up, the FPSS, 2000–08 (*N* = 26 913)

Characteristic	Total *N* (%)	Cases of mental ill-health *N* (%)	*P*-value
***Age (years)***			<0.0001
18–44	11 379 (42)	2176 (19)	
45–54	9614 (36)	1757 (18)	
55–69	5920 (22)	733 (12)	
***Married or cohabiting***			0.10
Yes	20 452 (76)	3507 (17)	
No	6461 (24)	1159 (18)	
***Occupational position***			0.80
High	7373 (27)	1264 (17)	
Intermediate	15 995 (60)	2778 (17)	
Low	3545 (13)	624 (18)	
***Shift work***			0.80
No	17 497 (65)	3026 (17)	
Yes	9416 (35)	1640 (17)	
***Current smoking***			<0.0001
No	23 107 (86)	3885 (17)	
Yes	3806 (14)	781 (21)	
***Heavy drinking***			<0.0001
No	25 592 (95)	4377 (17)	
Yes	1321 (5)	289 (22)	
***Chronic illness***			0.06
No	23 193 (86)	3980 (17)	
Yes	3720 (14)	686 (18)	
***Body mass index***			
Mean (SD)	25.0 (4.2)	25.1 (4.3)	0.007

Figures are numbers (%) unless otherwise stated.

*P* values: differences between groups.

Table 2 presents the results from the logistic regression models examining the prospective association between physical activity and symptoms of mental ill-health in all women. After adjustment for age, occupational position and marital status, those women who reported very high levels of physical activity at baseline were less likely to experience new symptoms of mental ill-health than their least active counterparts (OR = 0.80; 95% CI: 0.71–0.90). Further adjustments for night/shift work, baseline health behaviours and health slightly attenuated this association (OR = 0.85; 95% CI: 0.75–0.95). There was a dose–response relationship (inverse) between physical activity and mental ill-health as intermediate and high levels of physical activity were also associated with a lower likelihood of future mental ill-health (fully adjusted linear trend *P* = 0.002).

**Table 2:** Odds ratios (95% confidence intervals) for associations of levels of physical activity at Time 1 (2000 or 2004) and mental ill-health at Time 2 (2004 or 2008) in women^a^, the FPSS, 2000–08

		Odds ratio for future mental ill-health	
	Total *N*/*N* GHQ cases	Model 1^b^	Model 2^c^
Physical activity levels			
<14 MET hours per week	5850/1099	1.00 (reference)	1.00 (reference)
14≥ MET hours per week <30	9596/1655	0.90 (0.82–0.98)	0.92 (0.84–1.00)
30≥ MET hours per week <60	8232/1381	0.85 (0.78–0.93)	0.88 (0.81–0.96)
≥60 MET hours per week	3235/531	0.80 (0.71–0.90)	0.85 (0.75–0.95)
*P* for linear trend^d^		<0.0001	0.002

^a^Excludes those who screened positively for mental ill-health at baseline and participants with missing data in any of the study variables.

^b^Adjusted for age, occupational position and marital status.

^c^Additionally adjusted for shift work, smoking, heavy drinking, body mass index and chronic illness.

^d^*P*-value for linear trend across the levels of the explanatory variable.

Table 3 shows that in the fully adjusted model, very high levels of physical activity were significantly associated with a decreased likelihood of mental ill-health in midlife (aged 45–54 years) women (OR = 0.81; 95% CI: 0.66–0.99), but not in younger or older women. Moreover, in this age group only, there was a significant linear trend between physical activity and mental ill-health, adjusted for all covariates. However, the age interaction was not significant (*P* = 0.8).

**Table 3:** Odds ratios (95% confidence intervals) for associations of levels of physical activity at Time 1 (2000 or 2004) and mental ill-health at Time 2 (2004 or 2008) in women by age group^a^, the FPSS, 2000–08

		Odds ratio for future mental ill-health	
Physical activity level	Total *N*/*N* cases of mental ill-health	Model 1^b^	Model 2^c^
18- to 44-year olds	11 379/2176		
<14 MET hours per week	2236/460	1.00 (reference)	1.00 (reference)
14≥ MET hours <30 per week	3719/722	0.93 (0.82–1.06)	0.94 (0.83–1.07)
30≥ MET hours <60 per week	3691/678	0.87 (0.76–0.99)	0.89 (0.77–1.01)
≥60 MET hours per week	1733/316	0.86 (0.73–1.01)	0.88 (0.75–1.04)
*P* for linear trend^d^		0.02	0.07
45- to 54-year olds	9614/1757		
<14 MET hours per week	2109/430	1.00 (reference)	1.00 (reference)
14≥ MET hours <30 per week	3591/657	0.88 (0.77–1.00)	0.90 (0.79–1.03)
30≥ MET hours <60 per week	2905/507	0.83 (0.72–0.96)	0.86 (0.75–1.00)
≥60 MET hours per week	1009/163	0.75 (0.62–0.92)	0.81 (0.66–0.99)
*P* for linear trend		0.002	0.02
55- to 69-year olds	5920/733		
<14 MET hours per week	1505/209	1.00 (reference)	1.00 (reference)
14≥ MET hours <30 per week	2286/276	0.85 (0.70–1.03)	0.89 (0.73–1.08)
30≥ MET hours <60 per week	1636/196	0.82 (0.69–1.02)	0.89 (0.72–1.11)
≥60 MET hours per week	493/52	0.70 (0.50–0.96)	0.77 (0.55–1.07)
*P* for linear trend		0.02	0.13

^a^Excludes those who screened positively for mental ill-health at baseline and participants with missing data in any of the study variables.

^b^Adjusted for age, occupational position and marital status.

^c^Additionally adjusted for shift work, smoking, heavy drinking, body mass index and chronic illness.

^d^*P*-value for linear trend across the levels of the explanatory variable.

Table 4 presents the relationship between the change in physical activity levels between Time 1 (2000 or 2004) and Time 2 (2004 or 2008) and mental ill-health at Time 2 (2004 or 2008). Increased physical activity was associated with a decreased likelihood of mental ill-health at follow-up in 45- to 54-year-old women (fully adjusted OR = 0.78; 95% CI: 0.67–0.90) and 55- to 69-year-old women (fully adjusted OR = 0.78; 95% CI: 0.62–0.97). No significant association was observed in younger women. The age interaction was significant (*P *< 0.01).

**Table 4:** Odds ratios (95% confidence intervals) for associations of change in physical activity levels between Time 1 (2000 or 2004) and Time 2 (2004 or 2008) and mental ill-health at Time 2 (2004 or 2008) in women by age group^a^, the FPSS, 2000–08

		Odds ratio for mental ill-health	
	Total *N*/*N* cases of mental ill-health	Model 1^b^	Model 2^c^
All women			
No change	14 396/2510	1.00 (reference)	1.00 (reference)
Decreased	6787/1252	1.05 (0.97–1.13)	1.07 (0.99–1.15)
Increased	5522/864	0.86 (0.79–0.94)	0.88 (0.81–0.95)
18- to 44-year olds			
No change	5721/1080	1.00 (reference)	1.00 (reference)
Decreased	3024/598	1.05 (0.94–1.18)	1.06 (0.95–1.19)
Increased	2559/481	1.00 (0.89–1.13)	1.01 (0.89–1.13)
45- to 54-year olds			
No change	5382/1018	1.00 (reference)	1.00 (reference)
Decreased	2368/452	1.01 (0.89–1.14)	1.03 (0.91–1.17)
Increased	1784/271	0.77 (0.66–0.89)	0.78 (0.67–0.90)
55- to 69-year olds			
No change	3293/412	1.00 (reference)	1.00 (reference)
Decreased	1395/202	1.16 (0.97–1.39)	1.19 (0.99–1.43)
Increased	1179/112	0.75 (0.60–0.93)	0.78 (0.62–0.97)

^a^Excludes those who screened positively for mental ill-health at baseline and participants with missing data in any of the study variables.

^b^Adjusted for age, occupational position and marital status.

^c^Additionally adjusted for shift work, smoking, heavy drinking, body mass index and chronic illness.

## Discussion

The results of this study with a large cohort of Finnish working women showed that physical activity was associated with a reduced future risk of mental ill-health. This protective effect remained after adjustments for socio-demographic, work-related and lifestyle factors, health conditions and body mass index. Our findings also demonstrated an inverse dose–response relationship between physical activity and likelihood of later symptoms of mental ill-health. These findings with working women are consistent with previous prospective studies that have reported a dose–response relationship between physical activity and risk reduction for depression in population samples.^8^^,^^9^

A combination of physiological, biochemical and psychosocial mechanisms have been proposed to explain why physical activity is beneficial for the prevention of mental ill-health.^7^^,^^8^ It has also been suggested that the social aspects of physical activity may be particularly beneficial for the mental health of women.^7^ The distraction and enjoyment usually involved in leisure-time physical activity have been proposed to explain why it is more consistently associated with mental health benefits than domestic or work-related physical activity.^23^

In addition, our findings revealed that mid-life and older women who reported increased levels of physical activity were at significantly less risk of later mental ill-health than those who did not increase physical activity. This was not evident for younger women. A distinguishing feature between the former groups and the younger women is that very few of the latter were likely to be in menopausal transition. Women in Western societies typically reach menopause between the ages of 45–54 (the age of our mid-life group), on average at the age of 51.^24^ The majority experience symptoms, commonly hot flushes, night sweats, sleep and mood disturbances.^25–27^ However, many women continue to experience symptoms into their 60s.^28^^,^^29^ Most (89%) of the women in the oldest age group were aged 55–60 and thus also likely to be experiencing menopausal symptoms. Working women report that symptoms that they attribute to menopause, including depressed mood, anxiety and lowered self-confidence are problematic for them at work.^30^ Evidence from randomized control trials shows that physical activity interventions reduce vasomotor symptoms and improve mood and psychological well-being in menopausal women.^31^ Various mechanisms have been proposed, including improvements in cardiorespiratory fitness, physical self-esteem, self-worth and self-efficacy.^32–35^

A novel feature of our study in comparison with previous studies is the inclusion of a measure of ‘very high’ levels of physical activity (≥60 MET hours per week, equivalent to more than 600 min of moderate intensity exercise or 300 min of vigorous intensity exercise per week). Our results indicated that very high levels of physical activity may be associated with a significantly decreased likelihood of later symptoms of mental ill-health in mid-life women, but this was not as evident in younger and older women. However, the age interaction was not significant and this finding requires further investigation.

### Strengths and limitations

The strengths of this study include its prospective design, data from a large occupational cohort, limited attrition and objective measures, some derived from reliable public health registers. Furthermore, the FPSS is an on-going cohort study allowing for the investigation of women in all age groups. As with the Australian Longitudinal Study on Women’s Health,^9^ a further strength is that it allows for exclusion of baseline cases of mental ill-health and adjustment for many potentially confounding variables, although not for genetic factors that might predict both physical activity and depression.^36^ A possible limitation is that data about chronic diseases were derived from national health registers on a limited set of diagnosed major conditions and did not include mild or undiagnosed conditions; thus the true prevalence of chronic illness may be underestimated. Participants were working women from the public sector. They may not be typical of the general population of adult women in terms of mental and physical health, or levels of physical activity.

One limitation of our study is that we measured physical activity by self-report. Observational and biomechanical measurements would provide more accurate assessment of physical activity. Techniques for addressing shortcomings in the measurement of physical activity have been suggested^37^^,^^38^ but are rarely employed in large scale studies. Finally, our study did not include measures of menopausal status or use of hormone replacement therapy (HRT). However, menopausal status would not have substantially clarified the role of physical activity in the alleviation of menopausal symptoms as women vary greatly in their experience of symptoms throughout menopausal transition. It is possible that some women were using HRT to alleviate symptoms, and thus had more energy and enthusiasm for exercise. A further possibility is that mid-life women who engaged in very high levels of physical activity were not beset by the additional obligations of many working women of this age: caring for children and elderly parents.

## Conclusions

The results of this prospective study overcome many of the methodological constraints of previous work. They confirm findings of an inverse dose–response relationship between physical activity and risk of later mental ill-health in women, and show the additional protective effects of increasing activity for mid-life and older women. The mechanisms that might explain why mid-life and older women demonstrated significantly enhanced protection from later mental ill-health requires further exploration, but may involve psychological and physiological changes associated with menopausal transition. The difficulties that menopausal symptoms cause for working life are one of the most common reasons cited by women for electing to take HRT.^30^ Both HRT and physical activity are known to reduce troublesome menopausal symptoms. However, many women are keen to explore non-pharmacological options.^31^ The results of this study add to the growing body of evidence for an inverse dose–response relationship between physical activity and mental health in women. Further research is required to explore optimal levels of physical activity for the mental health of women at different ages and to provide insight into underlying mechanisms.

## Authorship

A. Griffiths, A. Kouvonen and J. Vahtera conceptualized and designed the study. A. Kouvonen and J. Pentti performed the statistical analysis and A. Griffiths wrote the first draft of the article. All authors contributed substantially to interpreting the data and revising drafts of the article, and they approved the final version.

## Funding

The Finnish Public Sector Study is supported by the European Union (EU) Era-Age 2 program and the Academy of Finland (grants 264944 and 267727). A.K. is supported by the Economic and Social Research Council (ESRC) and Medical Research Council (MRC) funded UK Clinical Research Collaboration Centre of Excellence for Public Health (Northern Ireland) (grants RES-590-28-0001 and MR/K023241/1). M.K. is supported by an ESRC professorial fellowship and by the Finnish Work Environment Foundation. A.V. is supported by the Academy of Finland (grant 267172).

*Conflicts of interest*: None declared.

**Key points**

- Mental ill-health is a leading and increasing cause of disability worldwide, particularly for women. This prospective study found an inverse dose response relationship between level of physical activity and later risk of mental ill-health in working women.
- Results also showed that mid-life and older women who reported increased physical activity over time demonstrated a reduced risk of later mental ill-health in comparison with those that did not increase physical activity. Since this protective effect of increased physical activity did not hold for younger women, a possible role of physical activity in the alleviation of menopausal symptoms is suggested.
- The study adds to the growing body of evidence that physical activity protects against future risk of mental ill-health in women.
- Further research is required to explore optimal levels of physical activity for the mental health of women at different ages and to provide insight into underlying mechanisms.
